# Thickness profiles of the corneal epithelium along the steep and flat meridians of astigmatic corneas after orthokeratology

**DOI:** 10.1186/s12886-020-01477-y

**Published:** 2020-06-19

**Authors:** Jiaqi Zhou, Feng Xue, Xingtao Zhou, Rajeev Krishnan Naidu, Yishan Qian

**Affiliations:** 1 Department of Ophthalmology, Eye and ENT Hospital, Fudan University; Key Laboratory of Myopia of The State Health Ministry, 200031, 83 Fenyang Road, Shanghai, People’s Republic of China; 2 Sydney Eye Hospital, Sydney, NSW 2000 Australia

**Keywords:** Corneal epithelium, Fourier-domain optical coherence tomography, Orthokeratology, Toricity

## Abstract

**Background:**

The aim of this study was to investigate the changes in corneal epithelial thickness along the principle meridians of astigmatic corneas after six months of overnight spherical myopic orthokeratology (OK) lens wear.

**Methods:**

This is a prospective study. Fifty-seven subjects with up to 1.50 diopters (D) of corneal toricity wore spherical OK lenses for 6 months. Evaluations of OK lens fit, visual acuity, refractions and corneal toricity (CT) were performed. Fourier-domain optical coherence tomography (FD-OCT) was conducted to measure the corneal epithelial thickness (ET) along the principle meridians of corneal toricity over a diameter of 6 mm. The means of △ET of the same diameter at individual meridians (△ETSm and △ETFm) were calculated and compared.

**Results:**

Visual acuity and refraction improved significantly after OK lens wear. △ETFm thinned more than △ETSm (*P* = 0.027) at 1.5 mm in radius. △ETSm thickened more than △ETFm at 2.5 mm (*P* = 0.019) and 3.0 mm (*P* = 0.036).∣△ETSm - △ETFm∣ were significantly correlated with the baseline central CT at 2.0 mm, 2.5 mm and 3.0 mm. ∣△ETSm - △ETFm∣was significantly correlated with the baseline peripheral CT at 2.5 mm.

**Conclusions:**

Overnight wear of spherical OK lenses resulted in differential changes in the thickness profiles of the corneal epithelium between the steep and flat meridians in eyes with corneal toricity.

## Background

Orthokeratology (OK) is a contact lens technology that results in the temporary correction of myopia using specially designed reverse geometry rigid-gas-permeable (RGP) contact lenses that are worn during sleep. There has been a resurgence in clinical interest into OK over the past decade due to emerging evidence of its’ role in controlling the progression of myopia, particularly in Chinese children [1, 2]. Modern OK lenses are designed to apply positive pressure over the central cornea and negative pressure in the mid-periphery under the steeper secondary “reverse” curve of the contact lens. In doing so, it produces a flattening of the central corneal treatment zone that corrects the myopic refractive error by reducing the corneal power, and a steepening of the mid-peripheral cornea stabilizing the lens.

Although the mechanism by which OK lenses change the biomechanical and topographical properties of the cornea is still not fully understood, the reshaping of the corneal epithelium remains one of the most important theories. OK lenses appear to produce their biomechanical changes through thinning of the central corneal epithelium and thickening of the paracentral epithelium [3–5]. In eyes with corneal toricity, the application of OK lenses may lead to differential changes in corneal epithelial thickness along the steep and flat axes, due to the different pressures exerted by the lens on the cornea in each of these meridians. Currently, there are no studies investigating the reshaping of the corneal epithelium in eyes with corneal toricity. Corneal toricity exists in a significant proportion of the general population, with studies demonstrating that over 90% of the population aged 5 to 40 years have corneal toricity of ≥0.25 diopters of cylinder (DC) in their central corneas [6, 7]. In eyes with corneal toricity, significant asymmetry of the paracentral cornea has also been found in the chords of 5 to 8 mm [8, 9]. Moreover, studies [10, 11] have found that long-term wear of OK lenses can lead to changes in corneal toricity even after discontinuation of wearing; which may partly be due to the differential remodeling of the corneal epithelium along each of the different meridians. A recent study by our group found that long-term OK lens wear resulted in a decrease in the corneal power along the flatter meridian and an increase in corneal toricity after discontinuation of OK lens wear for 1 month [12].

Therefore, investigating the effects of OK lens wear on epithelial remodeling in eyes with corneal toricity, and the differences in epithelial thickness changes along the steep and flat corneal meridians may allow us to better understand the biomechanical effects on the cornea and the mechanism by which OK lenses produce their refractive correction.

This study used a Fourier-domain optical coherence tomography (FD-OCT) system [13] to automatically generate topography maps of corneal epithelial thickness in subjects who wore OK lenses for a period of 6 months. The epithelial thickness profiles were compared between the steep and flat axes of the cornea to determine if OK lens wear induces different biomechanical and thickness changes along the different axes and regions of the cornea.

## Methods

### Subjects

This was a prospective, longitudinal study conducted at The Eye and ENT Hospital of Fudan University in Shanghai, China between February 2016 and December 2016. The inclusion criteria for subjects were as follows: 1) age between 8 and 40 years, 2) myopic spherical refractive error between − 0.75 to − 5.00 diopters of sphere (DS) and with-the-rule (WTR) astigmatic refractive error less than 1.50 DC, 3) corneal toricity less than 1.5 DC and corneal elevation difference along the 8 mm chord of the two respective principal meridians of corneal toricity (CED) less than 40 μm, 4) radius of corneal curvature between 39.75 to 46.00 D (7.34 to 8.5 mm), 5) horizontal corneal diameter greater than 11.0 mm, 6) agreeable to wear OK lenses for more than 8 h during sleep, 7) and willingness to participate in the clinical trial and provide signed written consent. The parents of subjects younger than 18 years old signed the written informed consents prior to enrollment into this study.

The exclusion criteria included a history of RGP contact lens wear or any current ocular or systemic disease. The research described in this study adhered to the tenets of the Declaration of Helsinki and was approved by the ethics committee of the Eye and ENT Hospital of Fudan University.

### Study protocol

The OK lenses used for this study were of a spherical four-zone reverse-geometry design (Emerald series, Euclid, USA) made in a Boston XO material (Bausch + Lomb, USA). The lenses measured 10.6 to 10.8 mm in overall diameter, with a back optic zone (BOZ) of 6 mm in diameter and a central thickness of 0.22 mm. The reverse curve was 0.5 mm wide, the alignment curve was 1.2–1.4 mm wide and the peripheral curve was 0.5 mm wide.

Orthokeratology lenses were dispensed to be worn overnight and removed soon after eye opening in the morning. A good lens centration, as indicated by a bull’s eye pattern on corneal topography maps, was expected. Should significant lens decentration (greater than 1.0 mm) occur or the unaided visual acuity drop below 20/25 during follow-up visits, new lenses would be ordered until a good lens fit and centration were achieved and visual acuity restored to better than or equal to 20/25.

All subjects underwent a thorough contact lens follow-up examination including uncorrected and corrected distance visual acuities (UDVA and CDVA), objective and subjective refraction, corneal topography, optical coherence tomography (OCT) and slit-lamp biomicroscopy. Measurements were conducted in the morning within one hour of lens removal. The patients were followed 1 day, 1 week, 1 month, 3 months and 6 months after commencement of OK lens wear. The baseline and 6 months’ post-OK lens wear measurement results were analyzed for this study.

### Corneal topography

A Pentacam analysis system (Oculus GmbH, Wetzlar, Germany) was used for measurements of corneal curvature, elevation, corneal toricity (CT) and thickness. Pentacam imaging of the cornea was performed by the same experienced examiner and three measurements were averaged for each result. Only the scans marked “OK” by the instrument were saved and analyzed. Corneal powers from Simulated Keratometry (SimK), which were derived from the axial curvature map were used in the current study [14]. The steep (Ks) and flat (Kf) keratometry values and their axes were displayed corresponding to diameter, with diameters ranging from 1.0 to 8.0 mm centered on the corneal apex. The central CT was defined as (Ks-Kf) at 3 mm (1.5 mm in radius) and the peripheral CT was defined as (Ks-Kf) at 6 mm (3.0 mm in radius). The pre-OK corneal elevation map (front) was used to analyze the corneal elevation difference (CED) along the 8 mm chord of the two respective principal meridians of corneal toricity. CED values were determined by subtracting the average height along the steep meridian from the average height along the flat meridian.

### Optical coherence tomography

An FD-OCT tomography setup (RTVue S, Optovue, Inc., CA) with a corneal adaptor module was used to measure the central corneal epithelial thickness of chords with a diameter of 6 mm. A high magnification corneal lens adapter (CAM-L) with a pachymetry scan pattern (6 mm scan diameter, 8 radials, 1024 axial scans each, repeated five times) was used for imaging the cornea [5]. Subjects were asked to focus their vision on a provided fixation target. Images were acquired when the targeting windows demonstrated a specular reflex on the corneal apex, which indicated that the incident OCT beam was perpendicular to the corneal apex. Epithelial thickness was derived using an FD-OCT system calculating the distance between the air–tear interface (first curve) and the epithelium–Bowman’s layer boundary (second curve). Processing and verification of the OCT images were conducted as previously described by Li et al. [13] OCT calibration was performed as previously described by Kim and Ehrmann [15]. Repeatability of the OCT measurements was generally good, matching the findings demonstrated by Li et al. [13] All examinations were performed by the same examiner.

**Fig. 1:**
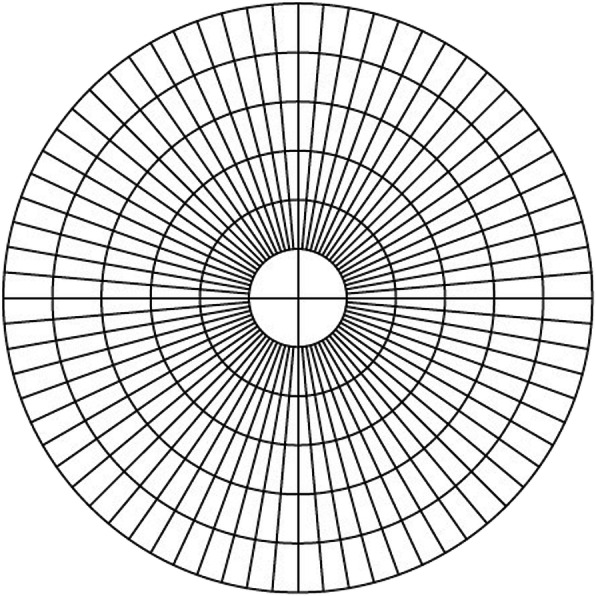
The transparency overlay with concentric circles used for the measurement of epithelial thickness

**Fig. 2:**
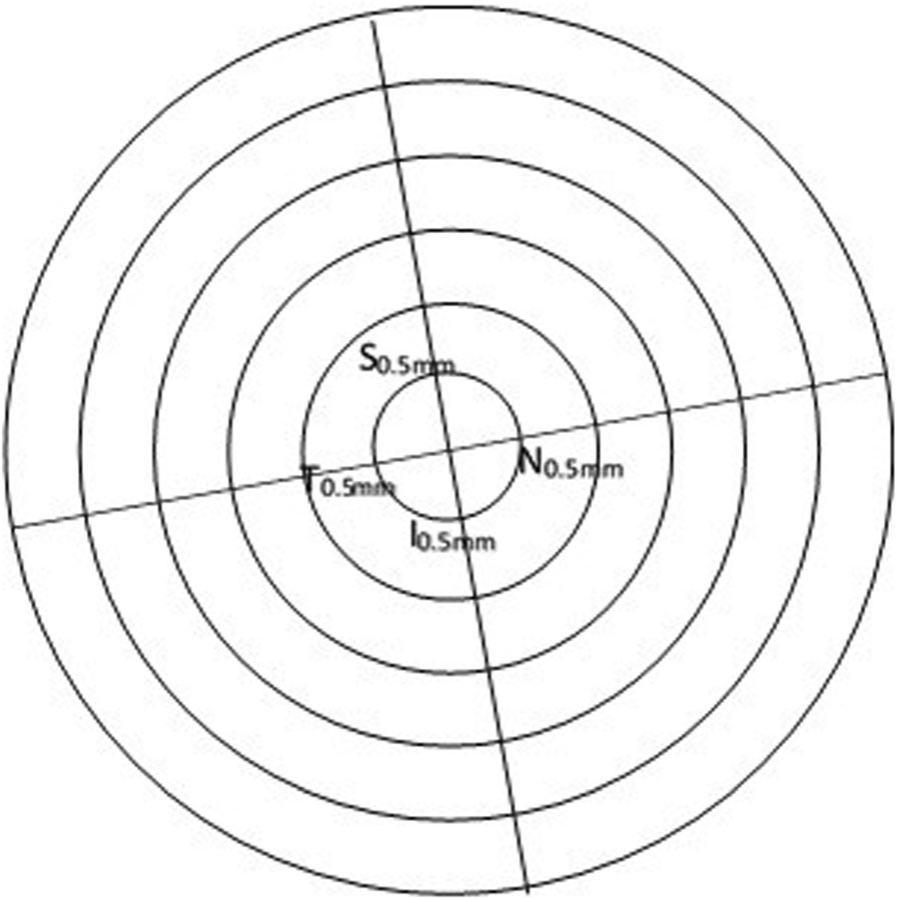
Illustration of the calculation of △ETSm and △ETFm

A 6 mm diameter epithelial thickness map was generated automatically. The observer placed a transparency with multiple concentric circles on top of the epithelial thickness map and situated the cursor on the individual point of interest (Fig. 1). The epithelial thickness map was divided into a central circle and 5 concentric annuli with an interval of 0.5 mm in radius (Fig. 2). The two principle meridians of central corneal toricity were marked on the epithelial thickness map and each meridian was divided into 12 segments with an interval step of 0.5 mm. Mean values of each segment were calculated using a custom-made software. The software collected several sample points from each segment and calculated the mean of these values after removing any outliers. The epithelial thickness along the steep (ETS) and flat (ETF) meridians were recorded and compared. The lens-induced changes in epithelial thickness (ET) were calculated as the difference (△ET) between the ET at 6 months post-OK lens wear and the baseline ET (6 m-pre). △ET of the steep meridian (△ETS) was compared to △ET of the flat meridian (△ETF). The means of △ET at the same radius along the same meridian (△ETSm and △ETFm, Fig. 2) was calculated; for example: △ETSm of 0.5 mm = (△ET of S_0.5mm_ + △ET of I_0.5mm_)/2; △ETFm of 0.5 mm = (△ET of N_0.5mm_ + △ET of T_0.5mm_)/2 (S: superior to apex, I: inferior to apex, N: nasal to apex, T: temporal to apex).

### Statistical analysis

Results for the descriptive statistics are presented as the mean ± standard deviation (SD). Simple comparisons between pre- and post-wearing were performed using Paired-t-test and comparisons between steep and flat axes using the Student’s t-test. The correlations between the different variables were studied using Pearson’s correlation coefficients. Probability values < 0.05 were considered statistically significant. Data analyses were performed using statistical analysis software (PASW 18.0, SPSS Inc., Chicago, IL).

## Results

The right eyes of 33 males (57.9%) and 24 females (42.1%) were included in this study. The mean age of all subjects was 13.6 ± 6.1 years (range: 9–38 years). The mean CED of 8 mm at baseline was 21.15 ± 8.79 μm (range: 0–39 μm). After 6 months of OK-lens wear, all subjects achieved good lens centration (decentration less than 1.0 mm), as indicated by a bull’s eye pattern on corneal topography maps. Unaided visual acuity achieved 20/25 or better in all of the subject eyes. The mean spectacle plane spherical error changed from − 2.77 ± 1.34 DS at baseline (range: − 5.00 to − 1.00 DS) to 0.13 ± 0.50 DS (range: 0.00 to + 1.50 DS, t = 13.87, *P* < 0.001) and the mean refractive astigmatism changed from − 0.45 ± 0.32 DC (range: − 1.25 to 0.00 DC) to − 0.49 ± 0.35 DC (range: − 1.50 to 0.00 DC, t = 0.802, *P* = 0.426). A summary of the refractive and topographic measurements is displayed in Table 1.

**Table 1:** Measurements of refraction, visual acuity, keratometry at baseline and after 6 months of OK lens wear

	Sphere, D*	RA, D	UDVA*	CDVA	Steep* Keratometry, D	Flat* Keratometry, D	Central CT (D)	Peripheral* CT (D)
Baseline	−2.77 ± 1.34	−0.45 ± 0.32	0.75 ± 0.22	−0.03 ± 0.04	43.90 ± 1.40	42.70 ± 1.10	1.06 ± 0.35	0.96 ± 0.36
6 month	0.13 ± 0.50	−0.49 ± 0.35	−0.04 ± 0.07	− 0.05 ± 0.06	41.90 ± 1.40	40.70 ± 1.40	1.17 ± 0.71	1.24 ± 0.56

D: diopters; RA: refractive astigmatism; SD: standard deviation; UDVA: uncorrected distance visual acuity; CDVA: corrected distance visual acuity; CT: corneal toricity; central CT: corneal toricity at 3 mm in diameter; peripheral CT: corneal toricity at 6 mm in diameter; * *P* < 0.05.

**Fig. 3:**
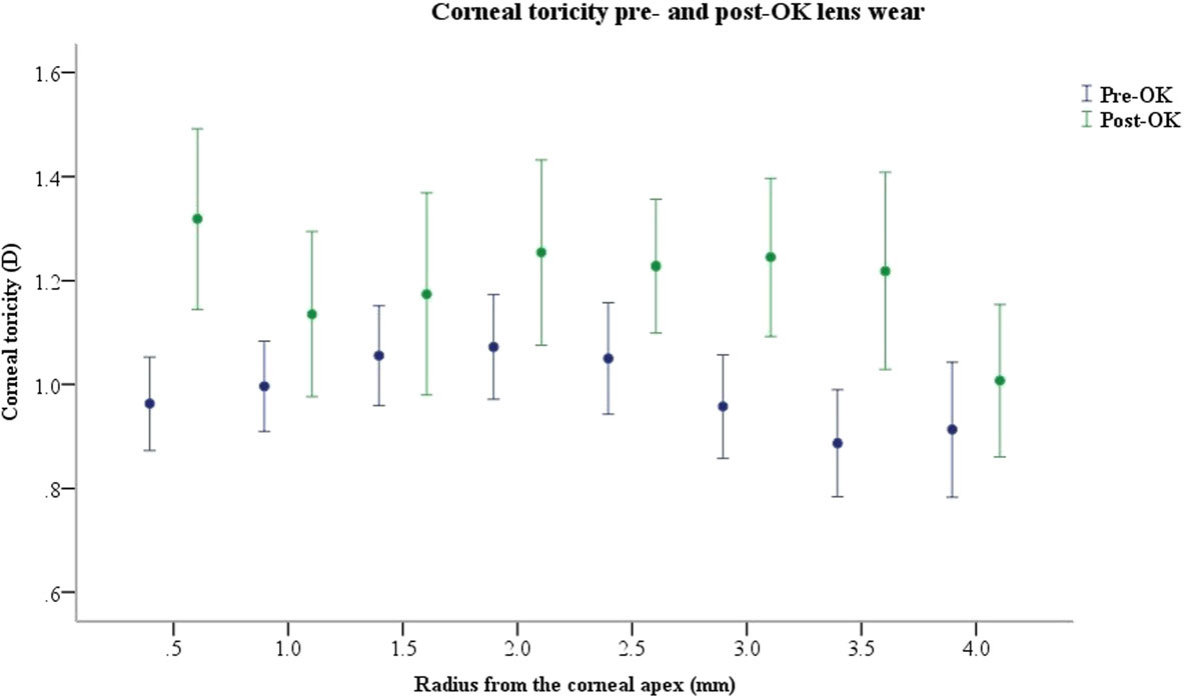
The distributions of corneal toricity according to the radius at baseline and 6 months post-lens wearing

The distribution of corneal toricity according to the radius at baseline and post-lens wearing is illustrated in Fig. 3. The differences in CTs between pre- and post-lens wear were significant for 0.5 mm, 2 mm, 2.5 mm, 3 mm, 3.5 mm and 4 mm in radius (Paired t test, P < 0.05).

**Fig. 4:**
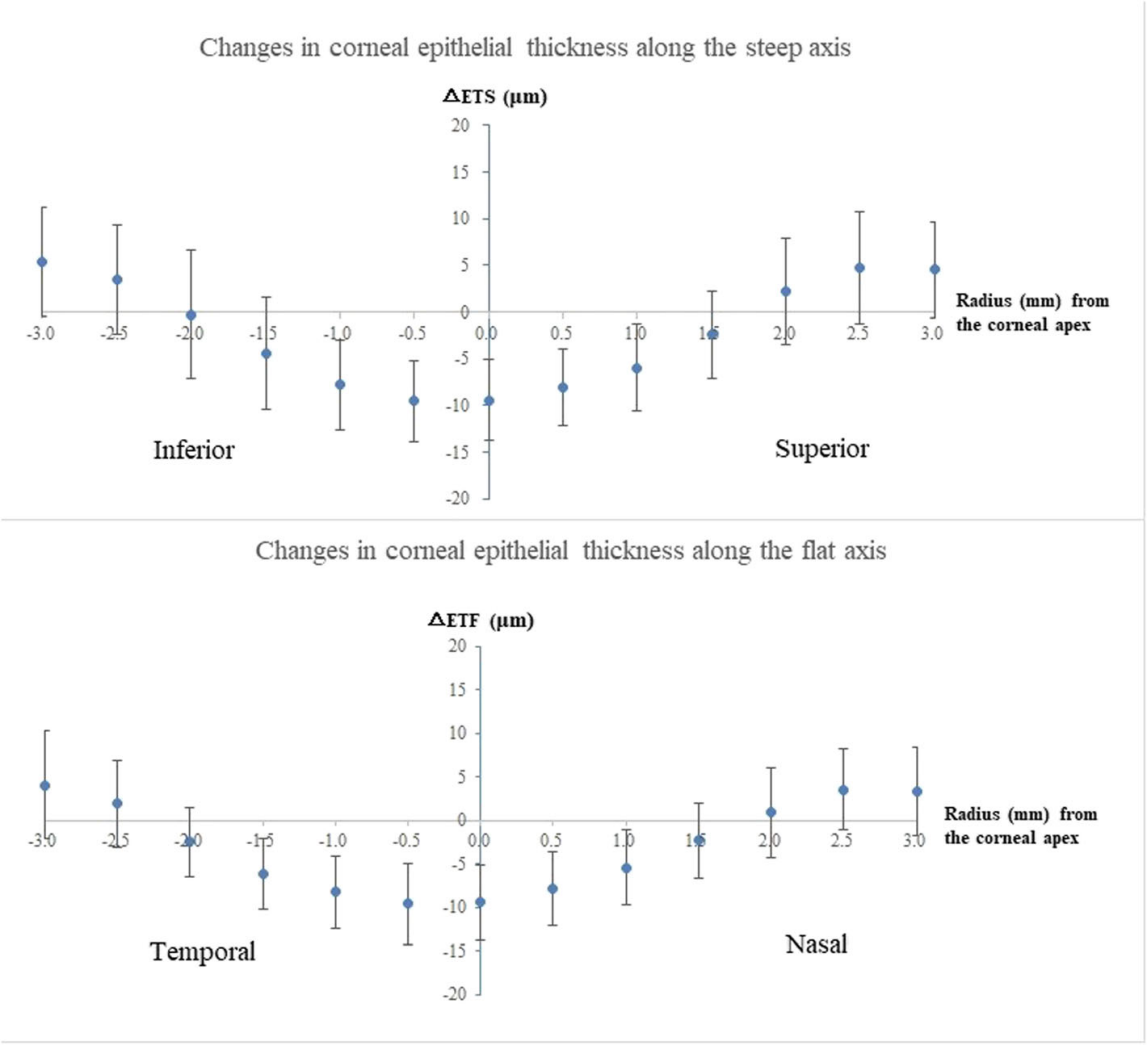
OK-lens induced changes in corneal epithelial thickness along the steep and flat axes. △ETS: changes in corneal epithelial thickness along the steep axis; △ETF: changes in corneal epithelial thickness along the flat axis

**Fig. 5:**
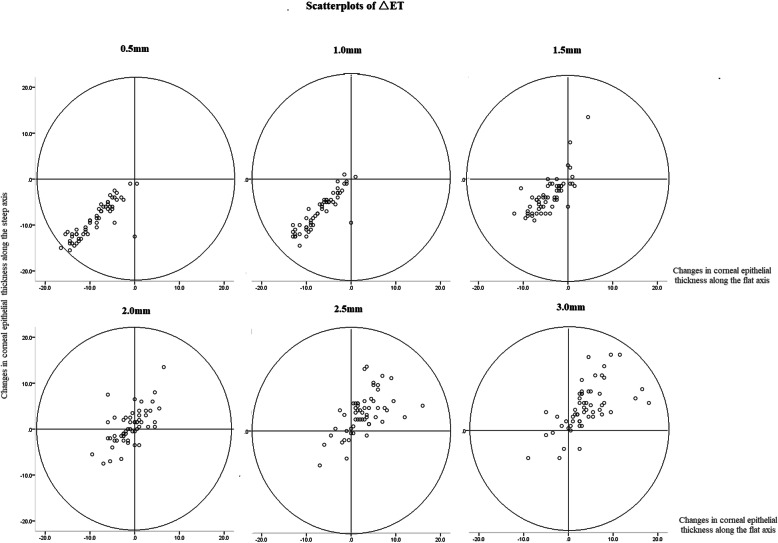
The scatterplots of △ET along the steep and flat axes in different radii. △ET: changes in corneal epithelial thickness

The central corneal epithelium thinned and the mid-peripheral corneal epithelium thickened after spherical OK lens wear (Fig. 4). Along the steep axis, the epithelium thinned most at 0.5 mm inferior to the corneal apex (− 9.2 ± 4.3 μm) and thickened most at 3 mm inferior to the apex (6.4 ± 6.4 μm). Along the flat axis, the epithelium thinned most at 0.5 mm temporal to the apex (− 9.2 ± 4.7 μm) and thickened most at 3 mm temporal to the apex (4.4 ± 5.9 μm). Figure 5 illustrates the scatterplots of △ET along the steep and flat axes in different radii.

**Fig. 6:**
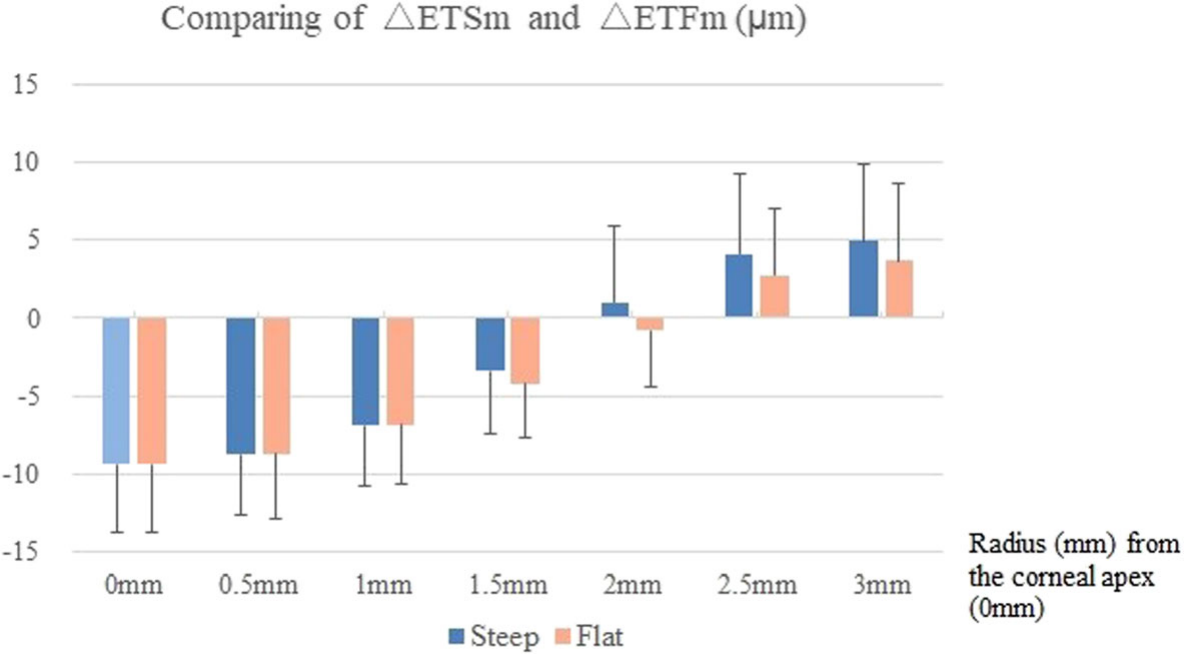
Comparison of the difference between △ETSm and △ETFm. △ETSm: the means of changes in corneal epithelial thickness along the steep axis; △ETFm: the means of changes in corneal epithelial thickness along the flat axis

In order to compare the differences in △ET between the steep and flat axes, the mean of the △ET (△ETm) at the same radius along each meridian was calculated and is illustrated in Fig. 6. No significant differences were found between △ETSm and △ETFm at 0.5 mm and 1 mm. △ETFm (− 4.2 ± 3.4 μm) thinned more than △ETSm (− 3.4 ± 4.0 μm, *P* = 0.027) at 1.5 mm. At 2.0 mm, △ETSm thickened, while △ETFm thinned (△ETSm: 1.0 ± 4.9 μm, △ETFm: − 0.8 ± 3.6 μm, *P* < 0.001). △ETSm thickened more than △ETFm at 2.5 mm (△ETSm: 4.1 ± 5.1 μm, △ETFm: 2.8 ± 4.2 μm, *P* = 0.019) and 3.0 mm (△ETSm: 5.0 ± 5.0 μm, △ETFm: 3.7 ± 4.9 μm, *P* = 0.036).

The absolute value of differences between △ETSm and △ETFm (∣△ETSm - △ETFm∣) were significantly correlated with the baseline central CT at 2.0 mm, 2.5 mm and 3.0 mm in radius (2.0 mm: r = 0.285, *P* = 0.032; 2.5 mm: r = 0.422, *P* = 0.001; 3.0 mm: r = 0.293, P = 0.027). ∣△ETSm - △ETFm∣was significantly correlated with the baseline peripheral CT at 2.5 mm in radius (r = 0.299, *P* = 0.028). No significant correlation was found between ∣△ETSm - △ETFm∣ and the post-OK lens wear central CT for any radius. ∣△ETSm - △ETFm∣was significantly correlated with the post-OK lens wear peripheral CT at 2.5 mm (r = 0.383, *P* = 0.004).∣△ETSm - △ETFm∣ was significantly correlated with baseline spherical refractive error at 2.0 mm, 2.5 mm and 3.0 mm (2.0 mm: r = 0.342, *P* = 0.009; 2.5 mm: r = 0.632, *P* < 0.001; 3.0 mm: r = 0.426, P = 0.001). ∣△ETSm - △ETFm∣was significantly correlated with baseline cylindrical refractive error at 2.5 mm (r = 0.303, *P* = 0.022).

## Discussion

Several mechanisms have been proposed to explain how OK lenses produce their refractive and myopia control effects, with changes in the thickness and topographical profiles of the corneal epithelium being one of the key mechanisms behind its’ action. In the current study, the OK lens-induced changes in the epithelial thickness profiles were evaluated and compared between the steep and flat meridians of corneas with toricity less than 1.50 diopters. After 6 months of overnight spherical myopic OK lens wear, our results demonstrated that the central corneal epithelium thinned and the mid-peripheral corneal epithelium thickened in all four quadrants. In the para-central cornea, ETF thinned more than ETS at 1.5 mm and 2 mm; in the mid-periphery, ETS thickened more than ETF at 2.5 mm and 3 mm. The differences between △ETSm and △ETFm were significantly correlated with the baseline central CT and the baseline peripheral CT. Our results suggest that there is a correlation between the corneal toricity and the remodeling of the corneal epithelium after wearing of OK lenses.

Thinning of the central corneal epithelium has been demonstrated in both clinical and histological studies of overnight OK lens wear [16, 17]; however, the reported changes in the mid-peripheral corneal epithelium have been less consistent. In a previous study by our research group [5], we found that the paracentral corneal epithelium thickened most in the inferior quadrant, which is similar to the findings of the present study and supported by the findings of a recent study by Reinstein et al. [3] Lian et al. [4] used a custom-made OCT to measure the thickness profile changes of the corneal epithelium after OK lens wear and found that the nasal and temporal mid-peripheral (4-6 mm) epithelium thickened after OK wear; however, they found no mid-peripheral epithelial thickening along the vertical meridian. They instead found that the epithelium tended to become thinner in the superior mid-peripheral region. They contributed this thinning of the superior mid-peripheral epithelium to greater pressure of the spherical OK lens on the superior cornea exerted by pressure from the upper eyelid, as well as superior decentration of the OK lens, which may have occurred in some of their subjects.

To the best of our knowledge, there are currently no clinical studies investigating the epithelial changes induced by spherical OK lens wear along different meridians of eyes with corneal toricity. Zhou et al. [18] compared the epithelial and stromal thicknesses along the steepest and flattest meridians between subjects with keratoconus and those with healthy corneas with corneal astigmatism ≥2 D. They found that the epithelium showed mid-peripheral thinning over the cone in addition to thickening superior to the cone in cases of keratoconus. Meanwhile, the average epithelial thickness map showed only small deviations in the distribution along the steepest meridian and the flattest meridian in healthy subjects. It was proposed that the epithelium compensates for anterior surface shape irregularities by altering its thickness profiles, which may be a similar mechanism observed with the ocular surface changes induced by OK lens wear. Investigating the effects of OK lens wear on epithelial remodeling in eyes with corneal toricity may allow us to better understand its biomechanical effects on the corneal profile. This current study found that ETF thinned more than ETS in the paracentral cornea, while ETS thickened more than ETF in mid-peripheral cornea. This could be explained by the mechanism by which spherical OK lenses reshape the cornea. The degree of pressure exerted by the OK lens depends on the sagittal height of the lens and the thickness of the tear film between the posterior lens surface and the cornea. The cornea along the steeper meridian is further away from the OK lens (i.e. greater sagittal height) than the flatter meridian. As a consequence, the thinning of the paracentral cornea is not as great along the steeper meridian as compared with the flatter meridian, and the thickening of the mid-peripheral epithelium is greater along the steeper meridian. The significant correlation between ∣△ETSm - △ETFm∣and baseline corneal toricity also helps to further support this mechanism. Although no significant correlation was found between ∣△ETSm - △ETFm∣and post-wearing central CT, ∣△ETSm - △ETFm∣was significantly correlated with the post-wearing peripheral CT at 2.5 mm. Therefore, the epithelial remolding could have an impact on the peripheral corneal toricity.

In theory, if following OK lens wear the epithelium thinned more along the flat meridian of the paracentral cornea and thickened more along the steep meridian of the mid-peripheral cornea, then the difference in corneal elevation between the flat and steep meridians should be reduced and the paracentral corneal toricity should also be reduced. However, we found an increase in the corneal toricity after OK lens wear. Previous studies [19, 20] have also indicated that OK lens wear could induce an increase in astigmatism, both regular and irregular. One possible explanation for this could be that the remodeling of the epithelium is neutralized by other factors such as lens decentration, uneven force imposed by the eyelids and stromal remodeling [19–21]. Further studies investigating the impact of corneal decentration on inducing corneal toricity are needed to clarify this issue.

Currently, most studies have only investigated the OK lens-induced epithelial changes along the horizontal meridian [20, 22]. Although Lian et al. [4] compared the thickness profile changes between the vertical and horizontal meridians, corneal toricity was not taken into account; and therefore, it is difficult to compare the findings of their study with those of the present study.

### Limitations of the study

Toric-designed OK lenses with toric peripheral curves were not included in this study. It was assumed that a toric OK lens would yield similar changes as spherical OK lenses since these lenses typically have a spherical optical zone. Further studies are warranted to investigate the effects of different OK lens designs on changes in the epithelial thickness profiles of astigmatic corneas. Also, this study only investigated the short-term results at a single time-point, with the data from before and after 6 months of OK treatment being analyzed. Data on how fast OK induces these ET changes and how long these changes remain after discontinuation of OK lens wear have not been explored currently, and further long-term studies with data at more frequent time intervals would help investigate this. Additionally, although the reverse curve of the OK lenses could exceed 6 mm, only the epithelial thickness of the central 6 mm could be obtained by the device used in the present study. Moreover, the axial resolution of the OCT used in this study is 5 μm, and therefore, for changes in epithelial thickness of less than 5 μm, the results are not as precise. These issues could be resolved by using an alternative instrument with more precise measurements and a greater range of analyses; however, these instruments are not yet available and are therefore not clinically relevant. Finally, the change in the thickness profile of the corneal stroma was not specifically measured in this study. It is possible that the long-term change in corneal toricity is a combination of changes in both the corneal epithelium and the stroma. Further studies with instruments that can measure the thickness profile of the corneal stroma are needed to clarify this question.

## Conclusions

Spherical OK lens wear results in greater thinning of the corneal epithelium along the flat meridian of the paracentral cornea, and greater thickening along the steep meridian of the mid-peripheral cornea. These findings add further evidence to the understanding of the mechanisms by which OK lens wear induces changes on the cornea and provides its refractive effects. In addition, the greater thinning of the epithelium along the flat meridian may help to explain the residual corneal flattening along the flat meridian after discontinuation of OK lens wear [10, 12].

## Data Availability

The datasets used and analysed during the current study are available from the corresponding author on reasonable request.

## Abbreviations

ETS: Corneal epithelial thickness along the steep axis
ETF: Corneal epithelial thickness along the flat axis
OK: Orthokeratology
CT: Corneal toricity
FD-OCT: Fourier-domain optical coherence tomography
ET: Epithelial thickness
△ETS: Change of ET at the steep meridian
△ETF: Change of ET at the flat meridian
DC: Diopters of cylinder
DS: Diopters of sphere
WTR: With-the-rule
BOZ: Back optic zone
UDVA: Uncorrected distance visual acuities
CDVA: Corrected distance visual acuities
Ks: Steep keratometry
Kf: Flat keratometry
△ETSm: The means of changes in corneal epithelial thickness along the steep axis
△ETFm: The means of changes in corneal epithelial thickness along the flat axis

## References

[1] Cho P, Cheung SW (2012). Retardation of myopia in Orthokeratology (ROMIO) study: a 2-year randomized clinical trial. Invest Ophthalmol Vis Sci.

[2] Hiraoka T, Kakita T, Okamoto F, Takahashi H, Oshika T (2012). Long-term effect of overnight orthokeratology on axial length elongation in childhood myopia: a 5-year follow-up study. Invest Ophthalmol Vis Sci.

[3] Reinstein DZ, Gobbe M, Archer TJ, Couch D, Bloom B (2009). Epithelial, stromal, and corneal pachymetry changes during orthokeratology. Optom Vis Sci.

[4] Lian Y, Shen M, Jiang J, Mao X, Lu P, Zhu D, Chen Q, Wang J, Lu F (2013). Vertical and horizontal thickness profiles of the corneal epithelium and Bowman’s layer after orthokeratology. Invest Ophthalmol Vis Sci.

[5] Qian Y, Xue F, Huang J, Qu X, Zhou X, Lanen-Wanek DV (2014). Pachymetry map of corneal epithelium in children wearing orthokeratology contact lenses. Curr Eye Res.

[6] Sanfilippo PG, Yazar S, Kearns L, Sherwin JC, Hewitt AW, Mackey DA (2015). Distribution of astigmatism as a function of age in an Australian population. Acta Ophthalmol.

[7] Leung TW, Lam AK, Kee CS (2013). Corneal shapes of Chinese emmetropes and myopic astigmats aged 10 to 45 years. Optom Vis Sci.

[8] Maseedupally V, Gifford P, Lum E, Swarbrick H (2013). Central and paracentral corneal curvature changes during orthokeratology. Optom Vis Sci.

[9] Chen Z, Xue F, Zhou J, Qu X (2017). Zhou X; Shanghai Orthokeratology and study (SOS) group. Prediction of orthokeratology lens decentration with corneal elevation. Optom Vis Sci.

[10] Wu R, Stapleton F, Swarbrick HA (2009). Residual corneal flattening after discontinuation of long-term orthokeratology lens wear in asian children. Eye Contact Lens..

[11] Santodomingo-Rubido J, Villa-Collar C, Gilmartin B, Gutiérrez-Ortega R (2014). Short-term changes in ocular biometry and refraction after discontinuation of long-term orthokeratology. Eye Contact Lens..

[12] Chen Z, Zhou J, Xue F, Zhou X, Qu X (2018). Increased corneal toricity after long-term orthokeratology lens wear. J Ophthamol.

[13] Li Y, Tan O, Brass R, Weiss JL, Huang D (2012). Corneal epithelial thickness mapping by Fourier-domain optical coherence tomography in normal and keratoconic eyes. Ophthalmology..

[14] Qian Y, Liu Y, Zhou X, Naidu RK (2017). Comparison of corneal power and astigmatism between simulated Keratometry, true net power, and Total corneal refractive power before and after SMILE surgery. J Ophthalmol.

[15] Kim E, Ehrmann K (2012). Assessment of accuracy and repeatability of anterior segment optical coherence tomography and reproducibility of measurements using a customized software program. Clin Exp Optom.

[16] Zhong X, Chen X, Xie RZ, Yang J, Li S, Yang X, Gong X (2009). Differences between overnight and long-term wear of orthokeratology contact lenses in corneal contour, thickness, and cell density. Cornea.

[17] Matsubara M, Kamei Y, Takeda S, Mukai K, Ishii Y, Ito S (2004). Histologic and histochemical changes in rabbit cornea produced by an orthokeratology lens. Eye Contact Lens..

[18] Zhou W , Stojanovic A . Comparison of corneal epithelial and stromal thickness distributions between eyes with keratoconus and healthy eyes with corneal astigmatism ≥ 2.0 D. PLoS One. 2014;9(1):e85994. 10.1371/journal.pone.0085994 PMC3904857 24489687

[19] Lian Y, Shen M, Huang S, Yuan Y, Wang Y, Zhu D, Jiang J, Mao X, Wang J, Lu F (2014). Corneal reshaping and wavefront aberrations during overnight orthokeratology. Eye Contact Lens.

[20] Hiraoka T, Okamoto F, Kaji Y, Oshika T (2006). Optical quality of the cornea after overnight orthokeratology. Cornea..

[21] Chen J, Huang W, Zhu R, Jiang J, Li Y. Influence of overnight orthokeratology lens fitting decentration on corneal topography reshaping. Eye Vis (Lond). 2018;5:5. 10.1186/s40662-018-0100-7 PMC5853138 29564358

[22] Alharbi A, Swarbrick HA (2003). The effects of overnight orthokeratology lens wear on corneal thickness. Invest Ophthalmol Vis Sci.

