# Correction to: Thickness profiles of the corneal epithelium along the steep and flat meridians of astigmatic corneas after orthokeratology

**DOI:** 10.1186/s12886-020-01607-6

**Published:** 2020-08-18

**Authors:** Jiaqi Zhou, Feng Xue, Xingtao Zhou, Rajeev Krishnan Naidu, Yishan Qian

**Affiliations:** 1 Department of Ophthalmology, Eye and ENT Hospital, Fudan University; Key Laboratory of Myopia of The State Health Ministry, 200031, 83 Fenyang Road, Shanghai, People’s Republic of China; 2 Sydney Eye Hospital, Sydney, NSW 2000 Australia

**Correction to: BMC Ophthalmol 20, 240 (2020)**

**https://doi.org/10.1186/s12886-020-01477-y**

Following publication of the original article [1], we were notified that the authors would like to remove affiliation 2 (Sydney Eye Hospital, Sydney, NSW, 2000, Australia) from the author Xingtao Zhou and Yishan Qian, and affiliation 1 (Department of Ophthalmology, Eye and ENT Hospital, Fudan University; Key Laboratory of Myopia of The State Health Ministry, 200031, 83 Fenyang Road, Shanghai, People’s Republic of China) from the author Rajeev Krishnan Naidu.
